# High Mortality Among Health Personnel With COVID-19 in Mexico

**DOI:** 10.1017/dmp.2020.382

**Published:** 2020-10-13

**Authors:** Irving Armando Domínguez-Varela

**Affiliations:** Tecnológico de Monterrey, School of Medicine and Health Sciences, Multicentric Ophthalmology Residency Program, Institute of Ophthalmology and Visual Sciences, Monterrey, México

**Keywords:** COVID-19, health personnel, Mexico, mortality, pandemic

On September 3, 2020, historical records of coronavirus disease (COVID-19) deaths among health personnel were published in Mexico with 1320 deaths, unlike the United States with only 1077.^1^ Approximately 1.8% in Mexico versus 0.54% in the United States of those killed by COVID-19 include health personnel (a little more than triple), representing an alert to doctors and health workers to look for the cause and with this, seek solutions.

The countries with the highest estimated numbers of health workers who have died from COVID-19 include Mexico (1320), United States (1077), UK (649), Brazil (634), Russia (631), India (573), South Africa (240), Italy (188), Peru (183), Indonesia (181), Iran (164), and Egypt (159).^1^

As of September 14, Mexico had one of the highest numbers of confirmed cases and deaths (668 381 and 70 821, respectively) in the region.^2^ Just 2 weeks ago (September 4), it was decided to enable the reopening of movie theaters, gyms, and casinos while respecting the health measures imposed by protocols. To date, some shopping malls and restaurants have been closed for not controlling the excessive number of people who have come to these places.

Comorbidities, such as diabetes and hypertension, are associated risks of poor outcomes in COVID-19 patients, with a prevalence of 18% and 32% in severe COVID-19, respectively. There’s a higher risk of intensive care unit (ICU) admission and mortality for patients with diabetes or hypertension who develop COVID-19.^3^ Obesity also represents a risk factor for ICU admission, severe COVID-19, and disease progression in patients with COVID-19.^4^ Applying these data in our country, we know that in Mexico there is a prevalence of 13.7% for diabetes, 25.5% for high blood pressure, and 31.6% for obesity.^5-7^ This puts Mexico above the world prevalence with 8.7%, 26%, and 13%, respectively.^8-10^ The prevalence of these comorbidities could explain why Mexico is the first country in mortality of health workers due to COVID-19.

The high mortality of health personnel in Mexico can be due to many factors; among the most important could be the prevention measures and lack of personal protective equipment provided to us and the higher prevalence of comorbidities compared to that of the rest of the world. It can also be attributed to the attitude of the population to this pandemic and the correct use of protection measures.

This pandemic took everyone by surprise and has shown us how vulnerable our health system is at the national level. Nevertheless, the government has to reactivate the economy by allowing the opening of businesses; however, the health personnel (nurses, doctors, technicians, workers in the COVID-19 area) are at a greater risk of complications in this country compared with other countries.^1^ “It’s like we are living in two different countries, in two different Mexicos, where we doctors feel at war, but the others feel that we have already emerged from this pandemic.”

Mexicans must work together with the government and the health ministry to get ahead of this crisis and prevent each other from going separate ways in this never-ending pandemic.
